# Dual Platform Long-Read RNA-Sequencing Dataset of the Human Cytomegalovirus Lytic Transcriptome

**DOI:** 10.3389/fgene.2018.00432

**Published:** 2018-09-27

**Authors:** Zsolt Balázs, Dóra Tombácz, Attila Szűcs, Michael Snyder, Zsolt Boldogkői

**Affiliations:** ^1^Department of Medical Biology, Faculty of Medicine, University of Szeged, Szeged, Hungary; ^2^Department of Genetics, School of Medicine, Stanford University, Stanford, CA, United States

**Keywords:** human cytomegalovirus, transcriptome, long-read sequencing, nanopore sequencing, CapSeq, Iso-Seq, direct RNA sequencing, Sequel

## Introduction

RNA-sequencing has revolutionized transcriptomics and the way we measure gene expression (Wang et al., 2009). As of today, short-read RNA sequencing is more widely used, and due to its low price and high throughput, is the preferred tool for the quantitative analysis of gene expression. However, the annotation of transcript isoforms is rather difficult using only short-read sequencing data, because the reads are shorter than most transcripts (Steijger et al., 2013). Long-read sequencing, on the other hand, can provide full contig information about transcripts, including exon-connectivity, and its merits in transcriptome profiling are being increasingly acknowledged (Sharon et al., 2013; Abdel-Ghany et al., 2016; Wang et al., 2016; Kuo et al., 2017). Due to the relatively low throughput of current long-read sequencing technologies, they can only characterize smaller transcriptomes in high-depth (Weirather et al., 2017).

The Human cytomegalovirus (HCMV) is a ubiquitous betaherpesvirus, which can cause mononucleosis-like symptoms in adults (Cohen and Corey, 1985), and severe life-threatening infections in newborns (Wen et al., 2002). Latent HCMV infection has recently been implicated to affect cancer formation (Dziurzynski et al., 2012; Jin et al., 2014). Examining the transcriptome of the virus can go a long way in helping understand its molecular biology. Short-read RNA sequencing studies have discovered splice junctions and non-coding transcripts (Gatherer et al., 2011) and have shown that the most abundant HCMV transcripts are similarly expressed in different cell types (Cheng et al., 2017). Our long-read RNA sequencing experiments using the Pacific Biosciences (PacBio) RSII platform revealed a great number of transcript isoforms, polycistronic RNAs and transcriptional overlaps (Balázs et al., 2017a).

### Data

**Figure 1:**
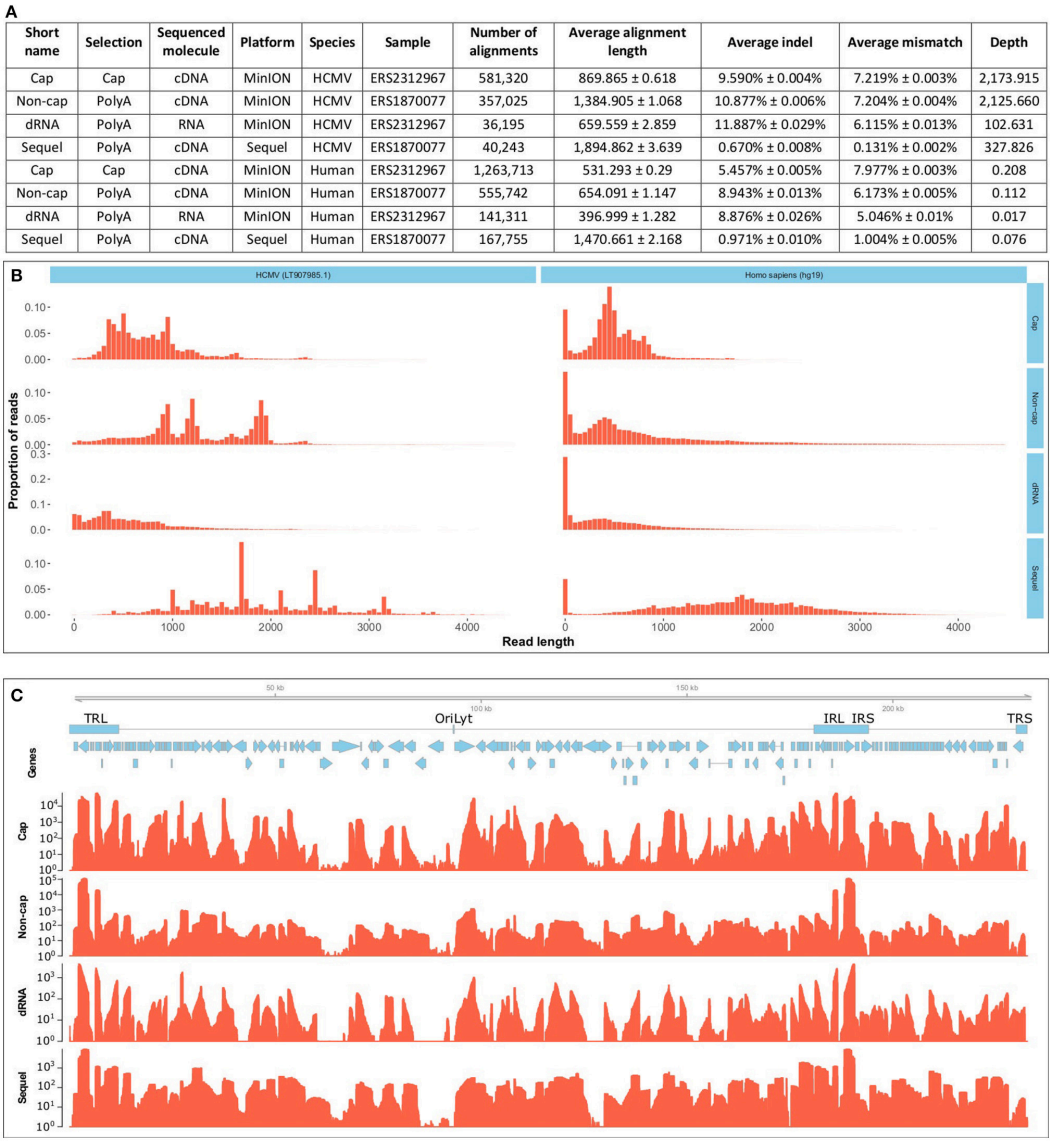
Data quality and metrics. **(A)** Summarizes the quality metrics of the sequencing reads for each sequencing run broken down per species (host and virus). All errors are given as standard errors. **(B)** Depicts the read-length distribution. The files have been uploaded to the ENA under the study accession PRJEB25680. Aligned read length is expressed in base pairs and the distribution is depicted for 50-basepair-long bins. The x axis is only presented until 4500 base pairs, even though the longest read was substantially longer (12079 bp), over 99% of the alignments fall into this range. The MinION platform generated predominantly shorter reads, than the Sequel platform. The non-cap selected cDNA library was selected for fragments longer than 500 bp. Cap, Cap-selected cDNA sequencing on the MinION platform; Non-cap, Non-cap selected cDNA sequencing on the MinION platform; dRNA, direct RNA sequencing on the MinION platform. Sequel, cDNA sequencing on the Sequel platform. **(C)** shows the depth of sequencing along the HCMV genome. Reads were aligned to the HCMV strain Towne genome (LT907985.1). Below the genome, blue rectangles denote the main repeat regions of HCMV. TRL, Terminal Repeat Long; OriLyt, lytic origin of replication; IRL, Internal Repeat Long; IRS, internal Repeat Short; TRS, Terminal Repeat Short. Blue arrows mark the CDS sequences of HCMV genes. The coverage is shown on a logarithmic scale for each sequencing method. Cap, Cap-selected cDNA sequencing on the MinION platform; Non-cap, Non-cap selected cDNA sequencing on the MinION platform; dRNA, direct RNA sequencing on the MinION platform. Sequel: cDNA sequencing on the Sequel platform. All methods included polyA-selection.

Here, we present the dual-platform long-read RNA sequencing dataset of two HCMV-infected fibroblast samples. We have sequenced the same RNA population that we have previously sequenced with the PacBio RS II platform (Balázs et al., 2017b), but now using the PacBio Sequel and Oxford Nanopore Technologies (ONT) MinION platforms. These data, apart from providing a more profound picture of the lytic HCMV transcriptome, can also be used to compare the current technologies. A further sample was prepared, using lytic HCMV RNAs. This sample was subjected to ONT Cap-selected cDNA sequencing (Cap-Seq) in order to allow better characterization of the transcription start sites, and also to direct (d)RNA sequencing in order to avoid reverse-transcription (RT) and PCR artifacts. We report of sequencing of approximately 100 GB raw data (Supplementary Table 1). The CapSeq by the MinION platform yielded the highest read count, the throughputs of the Sequel platform and the ONT dRNA sequencing both lagged behind (summarized in Figure 1A); both technologies nonetheless offer significant benefits. The Sequel platform is more accurate and the dRNA sequencing is free of RT and PCR artifacts. The read length distribution shows that the Sequel platform has a similar molecule-size preference to the RSII platform, while the MinION platform sequences more short reads (Figure 1B). The length-distribution of the non-cap selected cDNA sequencing reads are different from the other ONT reads, because this library was size-selected (>500 nt).

Each experiment shows a different coverage pattern along the HCMV genome (Figure 1C), which can be partly attributed to (1) whether or not cap-selection was applied, (2) whether or not the sample was reverse transcribed and amplified, (3) the length-preference of the platform, and (4) to the variance between the samples.

## Materials and methods

### Samples

**Figure 2:**
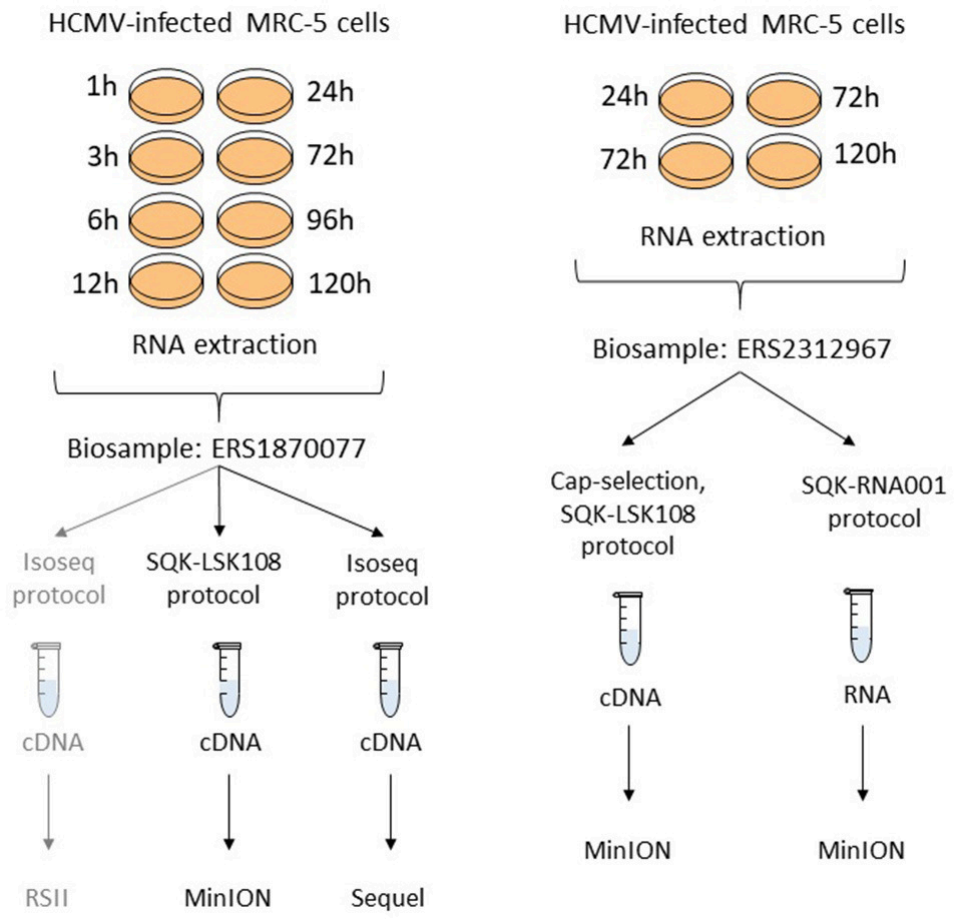
Experimental layout. Isolated RNA samples from different post-infection time points were pooled into two independent biosamples (accessions: ERS1870077 and ERS2312967). Different selection and library preparation protocols were used for each sequencing. The gray path denotes a previously published experiment (Balázs et al., 2017b).

Two independent biological samples (with Biosample accession numbers ERS1870077 and ERS2312967) were used in this study. The layout of the experiments has been summarized in Figure 2.

#### Biosample ERS2312967

Four T75 cell culture flasks (Thermo Fischer) of human lung fibroblast cells [MRC-5; American Type Culture Collection (ATCC)] were cultured at 37°C and 5% CO_2_-concentration in DMEM supplemented with 10% fetal bovine serum (Gibco Invitrogen) and 100 units of potassium penicillin and 100 μg of streptomycin sulfate per 1 ml (Lonza). Rapidly-growing near-saturated cell cultures were infected with HCMV strain Towne VarS (ATCC), at a multiplicity of infection (MOI) of 0.5 plaque-forming units (pfu) per cell. The infected cells were incubated for 1 h, after which the virus suspension was removed and washed with PBS. Following the addition of fresh culture medium, the cells were incubated for 24, 72, or 120 h (in 1-2-1 flasks, respectively). Total RNA was isolated from each sample using the NucleoSpin RNA kit (Macherey-Nagel) and 20 μl of each were pooled before reverse transcription.

#### Biosample ERS1870077

The same total RNA sample that had been sequenced and presented in our earlier publication (Balázs et al., 2017a) was also sequenced by Oxford Nanopore cDNA sequencing and the novel, high-throughput sequencing platform of Pacific Biosciences called Sequel. Briefly, pooled RNA sample was obtained from HCMV strain Towne VarS (ATCC) infected MRC-5 (ATCC) cells, that were grown under the same conditions as mentioned above. The infection was carried out at a MOI of 0.05 pfu per cell. Total RNA was isolated from infected cells at 1, 3, 6, 12, 24, 72, 96, 120 h post-infection (p.i.).

### Selection and library preparation

The Oligotex mRNA Mini Kit (Qiagen) was used to select polyadenylated RNAs from both samples. Four different, poly(A)-selected libraries were prepared in order to better characterize the HCMV transcriptome.

#### Direct RNA library for sequencing on the ONT platform

500 ng polyA-selected RNA was used from biosample ERS2312967 for direct RNA sequencing. A first-strand cDNA was synthesized using SuperScript IV (Thermo Fischer Scientific) and the adapter primers provided by the Direct RNA Sequencing kit (SQK-RNA001, Oxford Nanopore Technologies). The library was prepared using the Oxford Nanopore Ligation Sequencing 1D kit (SQK-LSK108) following the instructions of the manufacturer.

#### Non cap-selected cDNA library for sequencing on the ONT platform

31 ng polyA(+) RNA of biosample ERS1870077 was reverse transcribed using SuperScript IV (Thermo Fischer Scientific) and adapter-linked oligod(T) primers, and 5′ adapter sequences with three O-methyl-guanine ribonucleotides (synthesized by Bio Basic) were ligated to allow for second-strand synthesis. The cDNA was amplified through 18 cycles using KapaHiFi DNA polymerase (Kapa Biosystems). The PCR products were separated on an UltraPure Agarose (Thermo Fischer Scientific) gel and cDNA fragments larger than 500 nt were isolated using the Zymoclean Large Fragment DNA Recovery Kit. The library was prepared using the Ligation Sequencing 1D kit (SQK-LSK108, Oxford Nanopore Technologies) and the NEBNext End repair / dA-tailing Module NEB Blunt/TA Ligase Master Mix (New England Biolabs) according to the manufacturers' recommendations.

#### cDNA library for sequencing on the sequel platforms

2 milligrams polyA(+) RNA from biosample ERS1870077 was reverse transcribed using SuperScript IV (Thermo Fischer Scientific) and anchored oligod(T) primers, following the PacBio Iso-Seq protocol. The cDNA was amplified using the Clontech SMARTer PCR. The cDNA sample was not fractionated according to size. The library was prepared with the SMRTbell DNA Template Prep Kit 2.0 and bound to MagBeads (MagBead Kit v2) for sequencing using the P6-C4 chemistry.

#### Cap-selected cDNA library for sequencing on the ONT platform

Two micrograms of total RNA of biosample ERS2312967 was used for first strand cDNA synthesis using the TeloPrime Full-Length cDNA Amplification Kit (Lexogen). The 5′ adapter was ligated to the DNA-RNA hybrid overnight at 25°C. Endpoint PCR was performed using the reagents supplied in the kit. The libraries for cDNA sequencing were prepared using the Ligation Sequencing 1D kit (SQK-LSK108, Oxford Nanopore Technologies) and the NEBNext End repair / dA-tailing Module NEB Blunt/TA Ligase Master Mix (New England Biolabs) according to the manufacturers' recommendations.

Sample concentration was determined using the Qubit (ds)DNA HS Assay Kit (Thermo Fisher Scientific).

### Sequencing

#### ONT

All three libraries were sequenced on R9.4 SpotON Flow Cells with a MinION DNA/RNA sequencing device. The sequencing runs were carried out using MinKNOW. Voltage levels were set and reset in line with the suppliers' recommendations. Base calling was performed using Albacore v1.2.6.

#### Sequel

The prepared library was sequenced on a single SMRT cell using the Sequel system. The length of the run was 10 h. Consensus sequences were generated using SMRT-Link v5.0.1 (Potter, 2016).

### Read processing

All sequencing reads were aligned to both the human genome (hg19 build) and the HCMV strain Towne VarS genome (LT907985.1) using GMAP (Wu and Watanabe, 2005). The mapped reads have not been trimmed and may therefore contain terminal poly(A) sequences or 5′ adapter sequences (AGAGTACATGGG in case of the Sequel, TGGATTGATATGTAATACGACTCACTATAG in the case of the CapSeq and TGCCATTACGGCCGGG in case of the not cap-selected cDNA sequencing). These sequences are usually soft clipped and can be used to determine read strandedness. Direct RNA sequencing reads do not contain 5′ adapters; read directions are determined by the sequencer as RNA molecules enter the nanopores with the polyA-tail first. Read statistics were calculated using custom scripts (doi: 10.5281/zenodo.1034511). The data metrics were visualized using the ggplot2 (Wickham, 2016) and the Bioconductor (Hahne and Ivanek, 2016) R packages.

### Data validation

The quantification of RNA and cDNA fractions was carried out using a Qubit (Life Technologies) fluorometer. In the case of the library preparation for the Sequel sequencing optimal conditions for primer annealing and polymerase binding were determined using PacBio's Binding Calculator in RS Remote. An Agilent 2100 Bioanalyzer (Agilent High Sensitivity DNA Kit) was used to measure the library sizes. The used samples had RNA Integrity Numbers greater than 9.5. In order to confirm that the sequenced virus is VarS from strain Towne, we have carried out PCR with primers probing the deleted segment and with primers designed to the two flanking sequences of the deletion in VarS as described in (Balázs et al., 2017a).

### Data re-use

The dataset contains RNA sequencing reads from various post-infection time points during the lytic infection of HCMV and can be used to detect transcript isoforms, polycistronic RNA molecules, transcriptional overlaps or transcript features such as transcriptional start sites, transcriptional end sites and splice junctions both in HCMV and in the human fibroblast cell culture. The raw data files can be used to improve base calling methods. The raw PacBio single-molecule real time sequencing data contains information about the polymerase kinetics, stored as IPD values. Raw reads are supplied in the company-standard raw data formats: unmapped bam files for the Sequel data and fast5 files for the nanopore data. Mapped binary alignment (bam) files from each of the raw datasets have also been uploaded to facilitate re-use. These files can be analyzed for example using samtools (Li et al., 2009), bedtools (Quinlan and Hall, 2010), or the Genome Analysis Toolkit (Van der Auwera et al., 2013). The dataset can be used to detect structural or single nucleotide variation or to test detection tools. The data generated with different platforms can be compared to analyze the differences in the performance of the platforms or to screen for platform-specific errors. The dataset on the native RNA sequencing can also be used to investigate epitranscriptomic modifications.

## Data availability statement

Raw and mapped data files have been uploaded to the European Nucleotide Archive under the accession number PRJEB25680 (https://www.ebi.ac.uk/ena/data/view/PRJEB25680). All data can be used without restrictions.

## Author contributions

DT carried out the experiments. ZBa and AS processed and analyzed the data. MS and ZBo conceived and supervised the project. ZBa and ZBo wrote the manuscript.

### Conflict of interest statement

The authors declare that the research was conducted in the absence of any commercial or financial relationships that could be construed as a potential conflict of interest.
